# 517. Subcutaneous Sarilumab for the Treatment of Hospitalized patients with Moderate to Severe COVID19 Disease: A Pragmatic, Embedded, Multi-Center Randomized Clinical Trial

**DOI:** 10.1093/ofid/ofab466.716

**Published:** 2021-12-04

**Authors:** Westyn Branch-Elliman, Ryan Ferguson, Gheorghe Doros, Patricia Woods, Sarah Leatherman, Judith Strymish, Rupak Datta, Rekha Goswami, Matthew Jankowich, Nishant Shah, Thomas H Taylor, Sarah T Page, Sara Schiller, Colleen Shannon, Cynthia Hau, Maura Flynn, Erika Holmberg, Karen Visnaw, Rupali Dhond, Mary Brophy, Paul Monach

**Affiliations:** 1 Veterans Affairs Boston Center for Healthcare Organization and Implementation Research, Boston, MA; 2 VA Boston Healthcare System, Boston, MA; 3 Boston University School of Medicine, Boston, MA; 4 Yale School of Medicine - Yale New Haven Hospital, West Haven, CT; 5 Togus VA Medical Center, Togus, ME; 6 Providence VA Medical Center, Providence, RI; 7 White River Junction VA Medical Center, White River Junction, Vermont; 8 VA Boston, Boston, Massachusetts; 9 VA Boston Healthcare Sustem, Boston, MA

## Abstract

**Background:**

The aim of this pragmatic, embedded adaptive trial was to measure the effectiveness of subcutaneous sarilumab in addition to an evolving standard of care for clinical management of inpatients with moderate to severe COVID-19 disease (NCT04359901). The study is also a real-world demonstration of the realization of a prospective learning healthcare system.

**Methods:**

Two-arm, randomized, open-label controlled 5-center trial comparing standard care alone to standard care (SOC), which evolved over time, with addition of subcutaneous sarilumab (200 mg or 400 mg anti-IL6R) among hospitalized patients with moderate to severe COVID-19 not requiring mechanical ventilation. The primary outcome was 14-day incidence of intubation or death. The trial used a randomized play-the-winner design and was fully embedded within the EHR system, including the adaptive randomization process.

**Results:**

Among 417 patients screened, 162 were eligible based on chart review, 53 consented, and 50 were evaluated for the primary endpoint of intubation or death ( >30% of eligible patients enrolled) (Figure 1). After the second interim review, the unblinded Data Monitoring Committee recommended that the study be stopped due to concern for safety: a high probability that rates of intubation or death were higher with addition of sarilumab to SOC (92.6%), and a very low probability (3.4%) that sarilumab would be found to be superior.

Figure 1. Key Study Milestones, Outcomes, and Adaptations

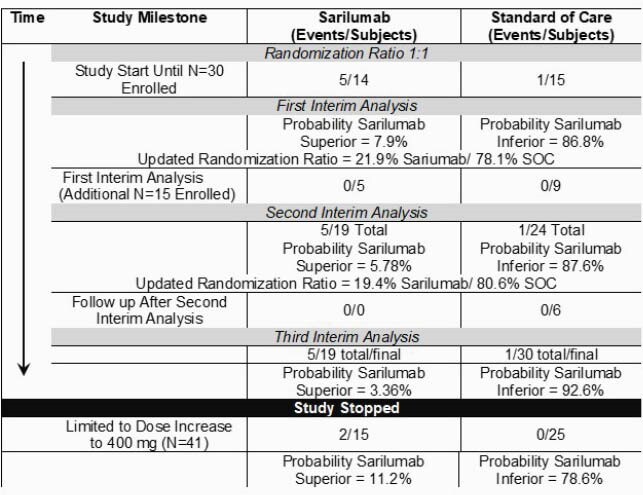

**Conclusion:**

This randomized trial of patients hospitalized with COVID-19 and requiring supplemental oxygen but not mechanical ventilation found no evidence of benefit from subcutaneous sarilumab in addition to an evolving standard-of-care. The numbers of patients and events were too low to allow independent conclusions to be drawn, but this study contributes valuable information about the role of subcutaneous IL-6 inhibition in the treatment of patients hospitalized with COVID-19. The major innovation of this trial was the advancement of embedded, point-of-care clinical trials for FDA-approved drugs; this represents a realization of the learning healthcare system. Methods developed and piloted during the conduct of this trial can be used in future investigations to speed the advancement of clinical science.

**Disclosures:**

**Nishant Shah, MD**, **General Electric** (Shareholder)**Pfizer, Inc.** (Research Grant or Support) **Karen Visnaw, RN**, **Liquidia** (Shareholder) **Paul Monach, MD,PhD**, **Celgene** (Consultant)**ChemoCentryx** (Consultant)**Kiniksa** (Advisor or Review Panel member)

